# Enhanced survival through repeated photodynamic therapy and almost complete tumor regression by prior radiation therapy in an orthotopic rat bladder cancer model

**DOI:** 10.1186/s12935-026-04465-2

**Published:** 2026-09-17

**Authors:** Mandy Berndt-Paetz, Sandra Nürnberger, Susann Gonsior, Ewelina Pączek-Hippe, Ina Patties, Annett Weimann, Romy Michalik, Jochen Neuhaus, Annegret Glasow

**Affiliations:** 1 Dept. of Urology, Research Laboratories, Leipzig University, Liebigstraße 19, Building C, 04103 Leipzig, Germany; 2 Institute for Pathology, Leipzig University, Liebigstraße 26, 04103 Leipzig, Germany; 3 Dept. of Radiation Oncology, Leipzig University, Stephanstraße 9a, 04103 Leipzig, Germany; 4 Comprehensive Cancer Center Central Germany (CCCG), Leipzig, Germany

**Keywords:** Photodynamic therapy, Near-infrared photosensitizer, Ionizing radiation, Synergistic cytotoxicity, Orthotopic rat bladder cancer model, Local immune cell recruitment

## Abstract

**Introduction:**

The development of new organ-preserving treatment alternatives is still an important issue in bladder cancer (BCa) research. A combination of minimally-invasive photodynamic therapy (PDT) with ionizing radiation (IR) could be beneficial for bladder tumor regression. Here, we tested the hypothesis that PDT with the near-infrared photosensitizer tetrahydroporphyrin-tetratosylate (THPTS) could treat bladder tumors safely and effectively in an orthotopic rat model.

**Materials and methods:**

Response additivity analysis of cytotoxicity was investigated in cultured tumor cells (AY-27) and spheroids consisting of AY-27 and bladder fibroblasts of F344 Fischer rats. For in vivo evaluation, tumors were induced in female Fischer rats by intravesical instillation of AY-27 cells. Triple-time treatments were performed, with ten rats each in four treatment groups: untreated controls (UTC), PDT, IR, PDT with prior IR (IR + PDT). Animals were euthanized when reaching the termination criteria, and bladders were evaluated by macroscopy, histology and CD45 immunohistology.

**Results:**

Synergism of combined treatment was demonstrated for all tested THPTS concentrations in AY-27/fibroblast spheroids but not in 2D cultured AY-27 cells. Tumor cell inoculation resulted in massive growth of solid bladder tumors (pT3a/b) in UTC. Triple-time treatment led to doubling of survival in the PDT group (mOS: 70 d) compared to UTC group (mOS: 36.5 d). IR monotherapy and IR + PDT resulted in complete survival without reaching the termination criteria. Histology revealed advantage of combination therapy vs. IR monotherapy by demonstrating treatment responses in all tumors of the IR + PDT group.

**Conclusion:**

This is the first in vivo study reporting doubling of survival time after PDT with a new near-infrared photosensitizer, and nearly complete tumor remission by neoadjuvant IR. This multimodal approach possibly allows minmally-invasive, organ-preserving BCa treatment.

**Supplementary Information:**

The online version contains supplementary material available at https://doi.org/10.1186/s12935-026-04465-2.

## Introduction

Bladder cancer (BCa) is number nine in the most common malignancies worldwide [1]. Non-muscle-invasive bladder cancer (NMIBCa, staged Ta, T1, Cis) is distinguished from muscle-invasive tumors (MIBCa, T2-T4). Standard treatment of NMIBCa includes transurethral resection followed by adjuvant chemo- or immunotherapy. Despite therapy, NMIBCa is frequently characterized by recurrences and progression to MIBCa [2]. In high-risk NMIBCa and MIBCa, radical cystectomy with postoperative chemotherapy is recommended. To date, there are no generally accepted options for an organ-preserving therapy of high-risk NMIBCa and MIBCa [3]. New therapeutic approaches to reduce recurrences and to provide bladder-preserving MIBCa treatment are urgently needed. Although recent therapeutic advances, including immune checkpoint inhibitors, fibroblast growth factor receptor inhibitors, and antibody–drug conjugates, have improved outcomes in selected patient subgroups, the prognosis for advanced disease remains poor, with limited overall survival [4]. Due to the good accessibility of the bladder, photodynamic therapy (PDT) is a promising option for minimally invasive treatment of urothelial cancer.

The principle of PDT is based on activation of a non-toxic photosensitizer (PS) by light of a specific wavelength leading to cellular damage by production of reactive oxygen species (ROS) and singlet oxygen (^1^O_2_). Two different types of reactions are known: Type I reaction leading to peroxides, hydroxyl radicals and superoxide ions via electron transfer cascades, and Type II reaction producing highly reactive singlet oxygen by direct energy transfer to ubiquitous molecular oxygen [5]. PDT destroys tumors directly by causing tumor cell death and indirectly via vascular shutdown and induction of an acute local inflammatory response resulting in immune system activation. Furthermore, immunogenic cell death triggered by PDT releases damage-associated molecular patterns that activate innate and adaptive immunity [6–8]. The photodynamic principle is implemented in diagnosis and therapies of several superficial epithelial tumor entities. Systemically and locally applied PSs (e.g. photofrin, haematoporphyrin derivates) have been used for PDT of NMIBCa in some clinical studies. However, systemic administration of PSs resulted in undesirable adverse effects such as skin photosensitization and local PDT caused bladder wall fibrosis due to high irradiation intensities used for activation [9]. Consequently, no standard PDT for superficial BCa could be developed, and PDT of muscle-invasive tumors is hampered by insufficient tissue penetration due to commonly applied light wavelengths of 630–690 nm reaching a light penetration of only up to 5 mm. PSs with maximal absorbance at higher wavelengths may increase the penetration depth.

Tetrahydroporphyrin-tetratosylat (THPTS) is a water-soluble, positively charged, near-infrared PS with an absorption maximum at 760 nm [10], allowing tissue penetration of up to 15 mm. In vitro studies already demonstrated the efficacy of THPTS-PDT in human retinoblastoma, glioblastoma and BCa cells [11–14]. Moreover, in BCa, single-time THPTS-PDT led to reduction of bladder tumor volumes 14 days after treatment in vivo [15]. THPTS preferentially accumulated into the lysosomes of bladder tumor cells and produced ROS after irradiation [14].

Radiotherapy with ionizing radiation plays an important role in trimodal therapy (TUR, radiotherapy, chemotherapy) as an organ-preserving alternative in MIBCa patients who are not candidates for cystectomy [16]. Like PDT, ionizing radiation (IR) generates free radicals and ROS, commonly resulting in cytotoxic effects like DNA damage, mitotic catastrophe, senescence and lipid peroxidation [17]. Therefore, the application of PDT in combination with IR seems to be a promising approach for anti-tumor treatment. Previous clinical studies demonstrated the advantage of PDT combined with radiotherapy in various cancer types including esophageal carcinoma and lung cancer [18]. In our recent in vitro study, the combinational therapy consisting of IR and THPTS-PDT led to multimodal cell death (via apoptosis, necroptosis, ferroptosis, pyroptosis), synergistic cytotoxicity and immune cell invasion in human bladder-like BCa organoids [19]. Here, we investigated the in vivo safety and efficacy of this novel bimodal strategy based on IR and transurethrally applied THPTS-PDT, which might extend the current standard therapy spectrum. The orthotopic AY-27/F344 Fischer rat bladder cancer model was used to examine the effects. In this well-characterized model, tumors can be induced by intravesical instillation of AY-27 BCa cells into the urinary bladder. The AY-27 cell line was initially derived from an N-(4-(5-nitro-2-furyl)-2-thiazylol) formamide (FANFT, 0.2%)- induced carcinoma of the urinary bladder in male Fischer rats [20]. We characterized concentration- and time-dependent cellular effects in 2D cultured AY-27 rat cells and spheroids. Furthermore, we analyzed survival rates, tumor regression and local immune stimulation in response to three cycles of IR + PDT compared to PDT or IR monotherapies in F344 Fischer rats.

## Materials and methods

### Animals

Female F344 Fischer rats (age: 9–14 weeks; weight: 150–180 g) were purchased from Janvier Labs (Le Genest-Saint-Isle, France). Animals were housed in groups of four rats per cage in a room with 12 h-inverted light/dark cycle and controlled temperature (22 ± 2 °C). Rat housing was performed in the Medical-Experimental Center, Medical Faculty, Leipzig University. Animal experiments were designed and performed in accordance with the guidelines of the European Communities Council Directive (2010/63/EU) and the German guidelines for the welfare of experimental animals (Tierschutzgesetz, TierSchG; Tierschutz-Versuchstierverordnung, TierSch-VersV). All experiments had been approved (Approval ID: TVV04/19 on 14 May 2019; T14/19 on 16 May 2019) in advance by the local authorities (Landesdirektion Sachsen).

### In vitro studies

#### Cell culture and organoid construction

Rat bladder carcinoma cells AY-27 were kindly provided by Prof. F. Guillemin and Dr. M.A. D´Hallewin (Centre de Lutte Contre le Cancer de Loraine, Nancy, France). Cell line authentication to confirm identity of AY-27 cultures was performed using the short tandem repeat (STR) profiling service supplied by Eurofins Genomics Europe (Ebersberg, Germany). Fibroblasts were primarily isolated by culturing fragments of de-epithelialized rat bladders of F344 Fischer rats in fibroblast-specific culture medium (FibroLife^®^ S2 medium, Cat.-No.: LL-0011, Lifeline Cell Technology, Frederick, USA) for 7 days. AY-27 cells were cultured in RPMI1640 medium supplemented with 10% FBS (Biochrom, Berlin, Germany) and 1% CTS™ GlutaMAX™-I Supplement (Cat.-No.: A1286001; Thermo Fisher Scientific, Dreieich, Germany). Cells were maintained at 37 °C and 5% CO_2_ in humidified atmosphere. To obtain single-cell suspensions, Accutase Cell Detachment Solution (Cat.-No.: P10-21500; PanBiotech, Aidenbach, Germany) was applied. AY-27 cells were characterized as poorly differentiated cells of urothelial origin (panCK, CK7, CK14: positive; CK13, CK20: negative; Additional file 1, A). The identity of bladder-derived fibroblasts (rBF) was confirmed by positive detection of vimentin and fibroblast marker TE-7 (Additional file 1, B).

The ultra-low attachment microplate method was used for spheroid formation. To generate BCa spheroids, AY-27 cells were mixed with rBF at equal quantities (6.000 cells each). The cell suspensions (12.000 cells per well) were centrifuged (250 x g; 5 min) into Corning^®^ Spheroid microplates (black; Cat.-No.: 4520; Corning Inc., Kennebunk, USA). AY-27/rBF spheroids were cultured in a 1:1 mixture of cell type-specific media until PDT and/or IR treatment.

#### Photodynamic therapy and ionizing radiation in vitro

Prior to treatment, AY-27 cells or AY-27/rBF spheroids were grown in black 96-well plates for 24 h (2D cell cultures) or 72 h (3D spheroids). Samples were treated with IR one hour before PS incubation using an orthovoltage X-ray machine (Xstrahl 200, Xstrahl, Ratingen, Germany). Irradiation was applied as single dose of 4 Gy (IR 4) with dose rates of 1.27–1.43 Gy/min (150 kV, 10 mA) after initial dose finding experiments using 2, 4, 6, and 8 Gy in CellTiter-Blue^®^Cell viability tests (Additional file 2, A). Sham-irradiated controls were shielded using MCP-96 blocks. THPTS (C_72_H_70_N_8_O_12_S_4_; MW: 1367.66 Da, purity ≥ 95%) was obtained from TetraPDT GmbH (Leipzig, Germany). The effectiveness of the photosensitizer THPTS was verified every three months by measuring its absorption spectrum and confirming the presence of the characteristic peak at 760 nm [10] using a SpectraMax M5 (Molecular Devices, Sunnyvale, USA). Samples were treated with 0, 6.25, 12.5, 25, 50 µM THPTS (light only control (LOC), PDT 6.25, PDT 12.5, PDT 25, PDT 50) freshly prepared from a 10 mM water stock solution, and diluted in culture medium. After 120 min of incubation, medium was replaced and cells/spheroids were irradiated at 760 nm using a diode laser (3 W, 760 ± 5 nm, LAMI-Helios, TetraPDT GmbH, Rackwitz, Germany). Laser light was delivered via a laser-coupled glass fibre producing a beam spot of 2 cm. Laser light intensity was measured and adjusted using a laser power meter (LaserStar, Ophir Optronics, Darmstadt, Germany). A light dose of 10 J/cm^2^ was delivered at an intensity of 0.8 W/cm^2^ for 12 s to individual wells as initially determined in irradiation dose finding experiments (Additional file 2, B). Non-irradiated samples were covered. After PDT, cells and spheroids were incubated for indicated time periods and assayed for cytotoxic effects.

#### Treatment response

After treatment, AY-27 cells and AY-27/rBF spheroids were incubated for additional 6, 24 and 72 h. Cell viability of 2D cultured AY-27 cells was determined via measurement of the metabolic activity using the CellTiter-Blue^®^ Cell Viability Assay (ex/em: 560/590 nm; Cat.-No.: G8080). Response additivity was used as reference model [21] to evaluate synergism of in vitro treatment. Expected effects of combination therapy were defined as sum of effects provoked by PDT or IR monotherapy alone (E_PDT_, E_IR_). For cell death mode, proportion of apoptotic cells was measured with the Apo-ONE^®^ Homogeneous Caspase-3/7 Assay (ex/em: 485/527 nm; Cat.-No.: G7790), and the loss of cell membrane integrity was analyzed by measurement of lactate dehydrogenase (LDH) release in the cell culture supernatant using the CytoTox-ONE™ Homogeneous Membrane Integrity Assay (ex/em: 560/590 nm; Cat.-No.: G7891). AY-27/rBF spheroid viability was determined using the CellTiter-Glo^®^ 3D Cell Viability Assay (Cat.-No.: G9681; Promega, Mannheim, Germany). All four assay types were obtained from Promega (Mannheim, Germany) and measured on a microplate reader (SpectraMax M5, Molecular Devices, Sunnyvale, USA). Spheroid morphology was analyzed by immunohistochemistry.

#### Immunohistochemistry

Spheroids were fixed by 4% paraformaldehyde and embedded into HistoGel (Cat.-No.: FIS12608606; ThermoFisher Scientific, Dreieich, Germany) 2, 16, 24, 72 h after treatment. After paraffin embedding, serial sections at 4 μm were prepared and deparaffinized, followed by antigen retrieval using heat-induced epitope retrieval buffer, pH 9.0 (Cat.-No.: ZUC029; Zytomed Systems, Berlin, Germany) for 40 min at 100 °C. After blocking of endogenous peroxidase, sections were treated with primary antibodies against 4-Hydroxynonenal (4-HNE; Cat.-No.: MAB3249, 1:20, R&D Systems, Abingdon, UK), phospho-histone H2A.X (p-H2A.X; Cat.-No.: #9718, 1:500, Cell Signaling Technol., Frankfurt/Main, Germany), cleaved caspase-3 (c-Casp3; Cat.-No.: #9664 1:500, Cell Signaling Technol., Frankfurt/Main, Germany) or cytokeratin AE1/AE3 (CK AE; Cat.-No.: MU071-UC, 1:200; BioGenex, Fremont, CA, USA) overnight at 4 °C. Biotin-linked secondary antibodies (goat-anti-mouse, goat-anti-rabbit; Vector Laboratories, Newark, USA) were incubated 45 min at room temperature. Following a coupling step with streptavidin-conjugated horseradish peroxidase, DAB was used for visualization and nuclei were counterstained with haematoxylin. Images were acquired using a Keyence BZ-X800 microscope (Keyence Coorporation, Osaka, Japan). BCa layer thickness and stroma core diameter of treated AY-27/rBF spheroids were measured using the Keyence BZ-X00 Analyzer software (Keyence Coorporation, Osaka, Japan).

### In vivo studies

**Fig. 1:**
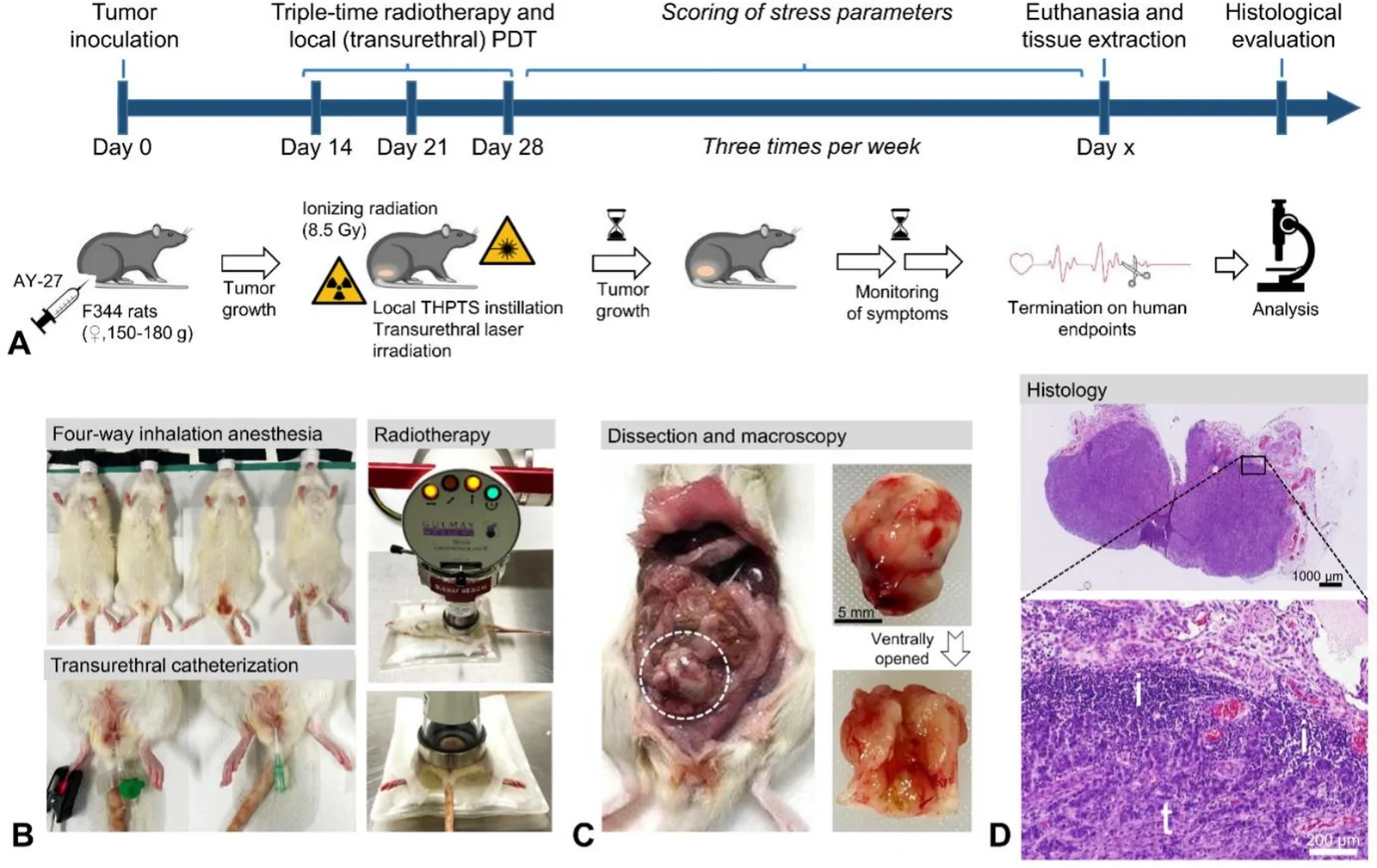
Time schedule. (**A**) Scheme of the workflow. Treatment was performed 14, 21, 28 days after tumor induction. Transurethral PS instillation was immediately conducted after radiotherapy. Animal welfare was monitored closely over the entire study period; animals were euthanized on reaching termination criteria. (**B**) Experimental setup. (**C**) Macroscopy of bladders after euthanasia; exemplarily shown for untreated control. Bladders were extracted, macroscopically documented and ventrally opened. (**D**) Microscopy of paraffin-embedded and HE-stained rat bladders. Tumor cell inoculation led to development of massively enlarged bladders with partially necrotic tumors infiltrating the perivesicular fat tissue; inflammation at the invasion margin was frequently observed (i, inflammation; t, tumor); bright red structures present large vessels at the tumor base

The applicability, safety, and efficacy of PDT with/without neoadjuvant IR was evaluated in vivo. An overview of the procedure and the experimental setup is given in Fig. 1.

#### Experimental cohorts

A preliminary study was conducted to assess the long-term outcome (trial period: 90 d) of single-time PDT (100 µM THPTS; 10 J/cm^2^). The THPTS concentration was selected based on our previous in vivo study, in which a single course of PDT monotherapy with 100 µM THPTS and irradiation at 10 J/cm² resulted in a significant reduction in tumor volume 14 days after treatment [15]. This dose was well tolerated; however, complete tumor remission was not achieved. We therefore hypothesized that combining this PDT with IR would produce an additive or synergistic antitumor effect. Preliminary experiments confirmed tolerability and effect of the IR dose (3 × 8.5 Gy) encompassing the whole bladder. A higher number of fractions was not feasible as animals had to undergo anesthesia for each irradiation. The applied dose corresponds to the biological effective dose (BED) of the currently recommended irradiation schedules for human BCa treatment (32 × 2 Gy or 20 × 2.75 Gy) [22] on late reacting normal bladder tissue (α/β: 3) and is about 65% of the BED in early reacting bladder tumor tissue (α/β: 10) (Table 1).

**Table 1:** BED dose calculations; based on [23, 24]

Dose/fraction (d)	Number of fractions (*n*)	α/β	$\:BED=nd(1+\frac{d}{{\upalpha\:}/\beta\:})$
**8**,**5**	**3**,**00**	**10**	**47**
2,75	20,00	10	70
2	32,00	10	77
**8**,**5**	**3**,**00**	**3**	**98**
2,75	20,00	3	105
2	32,00	3	107

Therefore, it can be assumed that the dose regimen applied here will produce effects on the normal bladder similar to those of the human schedule, while its antitumor efficacy still leaves room for enhancement through additional PDT effects. The main trial investigated the effects of multiple PDT ± prior IR versus monotherapies in terms of survival and tumor regression (Table 2).

**Table 2:** Experimental cohorts. PS, preliminary study; MS, main study; n_i_, number of inoculated rats; n_a_, number of rats included in survival and histopathological analysis

Trial	Experimental groups	*n*_i_	*n*_a_	Trial period (d)	End points
PS I: Single-time PDT	1 x UTC	12	12	85	*Primary end point*: Survival time;
	1 x PDT (100 µM THPTS, 10 J/cm^2^)	12	12	85	
PS II: Triple-time IR	3 x UTC	7	7	120	
	3 x IR (8.5 Gy)	5	5	120	
MS: Triple-time PDT ± prior IR	3 x UTC	10	10	85	*Secondary end point*: Tumor size
	3 x PDT (100 µM THPTS, 10 J/cm^2^)	10	7	85	
	3 x IR (8.5 Gy)	10	8	85	
	3 x IR + PDT	10	8	85	

#### Tumor inoculation

Tumors were induced as first established by Xiao et al. [20]. Using this method, we have demonstrated a 100% tumor induction rate in our previous study [15]. Female F344 Fischer rats were anesthetized using isoflurane inhalation anesthesia. For induction of anesthesia, rats were placed in a chamber supplied with 4% isoflurane (Isoflurane CP; WDT, Garbsen, Germany) in oxygen at a flow rate of 300 l/min. The animals were then transferred to a warming plate and inhalation anesthesia (100–200 l/min oxygen with 3% isoflurane) was administered directly via the nose and mouth. Four animals were treated simultaneously via separate breathing tubes. Pre-operative analgesia with metamizole (60–100 mg/kg diluted in 2 ml 0.9% NaCl) was given subcutaneously. Transurethral catheterization was done with an 18 G Insyte™ Autogard™ (Cat.-No.: 381847; BD, Franklin Lakes, NJ, USA) catheter. Bladder conditioning was performed by washing with 0.1 N HCL (0.5 ml) for 15 s and 0.1 N (0.5 ml) NaOH for 15 s, followed by five PBS washing steps. Tumor inoculation was done by instillation of 2 × 10^6^ AY-27 cells (in 0.5 ml culture medium) for 60 min. The animals were kept for 14 d until treatment.

#### Photodynamic therapy and ionizing radiation in vivo

The animals were treated three times at 7-day intervals. Cohorts consisting of four rats (one rat per treatment group: UTC, PDT, IR, IR + PDT) were treated in one experimental run. Animals were anesthetized i.m. with a mixture of 0.005 mg/kg fentanyl (Fentadon^®^ 0.05 mg/ml; Dechra Veterinary Products Deutschland GmbH, Aulendorf, Germany), 2.0 mg/kg midazolam (Dormicum^®^ 5 mg/ml; CHEPLAPHARM Arzneimittel GmbH, Greifswald, Germany) and 0.15 mg/kg medetomidine (Domitor^®^ 1 mg/ml; Orion Pharma GmbH, Hamburg, Germany). The rats were restrained on warming pads in supine position. A circular area (diameter: 3 cm) of the caudal abdomen was irradiated with 8.5 Gy at 11.6 Gy/min dose rate (150 kV, 20 mA) on a 200 kV orthovoltage X-ray generator (Gulmay D3225, Gulmay GmbH, Krefeld, Germany) (Fig. 1, B). Rats of the UTC and PDT group were also restrained in the supine position and sham-irradiated. The animals were then transferred onto a heating plate and anesthesia was prolonged using 1% isoflurane as described above. Pre-operative analgesia with metamizole (60–100 mg/kg, diluted in 2 ml 0.9% NaCl; WDT, Garbsen, Germany) was given subcutaneously. Transurethral catheterization was done with an 18 G Insyte™ Autogard™ (Cat.-No.: 381847; BD, Franklin Lakes, NJ, USA) catheter. Bladders were instilled with THPTS (100 µM in 0.9% NaCl, 0.5 ml) for 2 h. Untreated and IR-treated bladders were instilled with 0.9% NaCl. Following rinsing, bladders were filled with 0.9% NaCl and irradiated transurethrally via a glass fibre with laser light at 760 nm using a diode laser (3 W, 760 ± 5 nm, LAMI-Helios, TetraPDT GmbH, Rackwitz, Germany) and a light dose of 10 J/cm^2^ (0.06 W/cm^2^ for 3 min). After treatment, animals were kept under standard conditions until reaching the termination criteria or twice the survival time of the untreated controls.

#### Post-interventional monitoring and human end points

Orthotopic tumor growth did not allow continuous monitoring of the tumor size during the trial period. Nevertheless, close health monitoring and defined human endpoints eliminated high burden caused by the growing tumor. Animal welfare was daily inspected over the entire test period. Comprehensive scoring of the health condition (including body weight, body temperature, behavior, trial-specific stress parameters) was performed daily for the first two days after treatment and then three times per week. Metamizole (100 mg/kg; WDT, Garbsen, Germany) diluted in drinking water was administered for pain management during the entire trial period. Rats were immediately euthananized upon reaching any of the termination criteria (human endpoints) specified in the animal experiment proposal: (i) weight loss > 20%, (ii) apathy, abnormal body movement/position indicating pain, (iv) insufficient food and water uptake, (iii) macrohematuria, (v) persistent infections at injection sites (≥ 72 h). Animals that did not reach the endpoints were euthanized after twice the survival time of untreated controls. The time from day of tumor cell instillation to euthanasia (survival time) was analyzed for the different treatment groups using the Kaplan-Meier method. Median survival was defined as time point when half of rats in a group were still alive.

#### Bladder processing and evaluation

Bladders were excised and opened ventrally by a median incision. Metastatic disease was assessed by macroscopic examination of the kidneys, lungs, liver, spleen, and lymph nodes. After macroscopy, bladders were fixed (buffered formaldehyde 4% solution, pH 7.2; VWR, Darmstadt, Germany) for 24 h. Paraffin-embedded bladders were serially sectioned (7 μm). Every 20th section was stained with hematoxylin/eosin and analyzed for presence of viable tumors, maximum basal diameter of tumors, pT classification, and tumor regression grades by a pathologist using previously reported tumor regression grades [25, 26] with minor modifications (Table 3). Representative slices of bladder tumors corresponding to different regression grades were additionally stained by Crossmon´s trichrome.

**Table 3:** Tumor response grades; adapted with modification from Kim et al. [26]

Tumor regression grades (TRG)	Characteristics
No or minimal regression (NR)	Dominant tumor cell mass with obvious fibrosis or no regression
Moderate regression (MR)	Fibrotic changes with few tumor cells or groups
Near complete regression (NCR)	Singular islets of tumor cells invading the lamina propria
Complete regression (CR)	No residual cancer cells

Sections were further immunostained for abundance of leukocytes (CD45; Cat.-No.: ab10558, 1:400; Abcam, Cambridge, UK) and T cells (CD3; Cat.-No.: ab16669, 1:400; Abcam, Cambridge, UK) as decribed in Sect. 'Immunohistochemistry'. Images of HE-, Crossmon- and DAB-stained samples were acquired using a Keyence BZ-X800 microscope (Keyence Coorperation, Osaka, Japan). Maximum basal diameter of tumors was measured for quantification of tumor size using the Keyence BZ-X800 Analyzer software (Keyence Coorporation, Osaka, Japan). Local immune stimulation was analyzed in tumors by quantification of highly CD45/CD3-positive stained areas using the IHC profiler plugin [27] in Fiji, including ImageJ version 1.53 [28].

### Statistical analysis

Statistical analyses were performed using Prism 10.4.0 statistical software (GraphPad Software Inc., La Jolla, CA, USA). Bar diagrams of in vitro experiments present the means + standard deviation (SD) from at least three independent experiments (*n* = 3). Gaussian distribution of values was tested by the D´Agostino-Pearson omnibus normality test. Statistical differences in cell culture experiments were analyzed by One-way ANOVA, Dunnett´s multiple comparison test and Mann-Whitney test.

Weight curves of experimental animals present means ± SD. The Kaplan-Meier curves were statistically compared by the Gehan-Breslow-Wilcoxon test. Proportions of viable tumors within treatment groups were analyzed by single comparison using the Fisher´s exact test. *P* ≤ 0.05 was considered as statistically significant.

## Results

### Cellular effects in 2D cultured AY-27 cells and AY-27/rBF spheroids

**Fig. 2:**
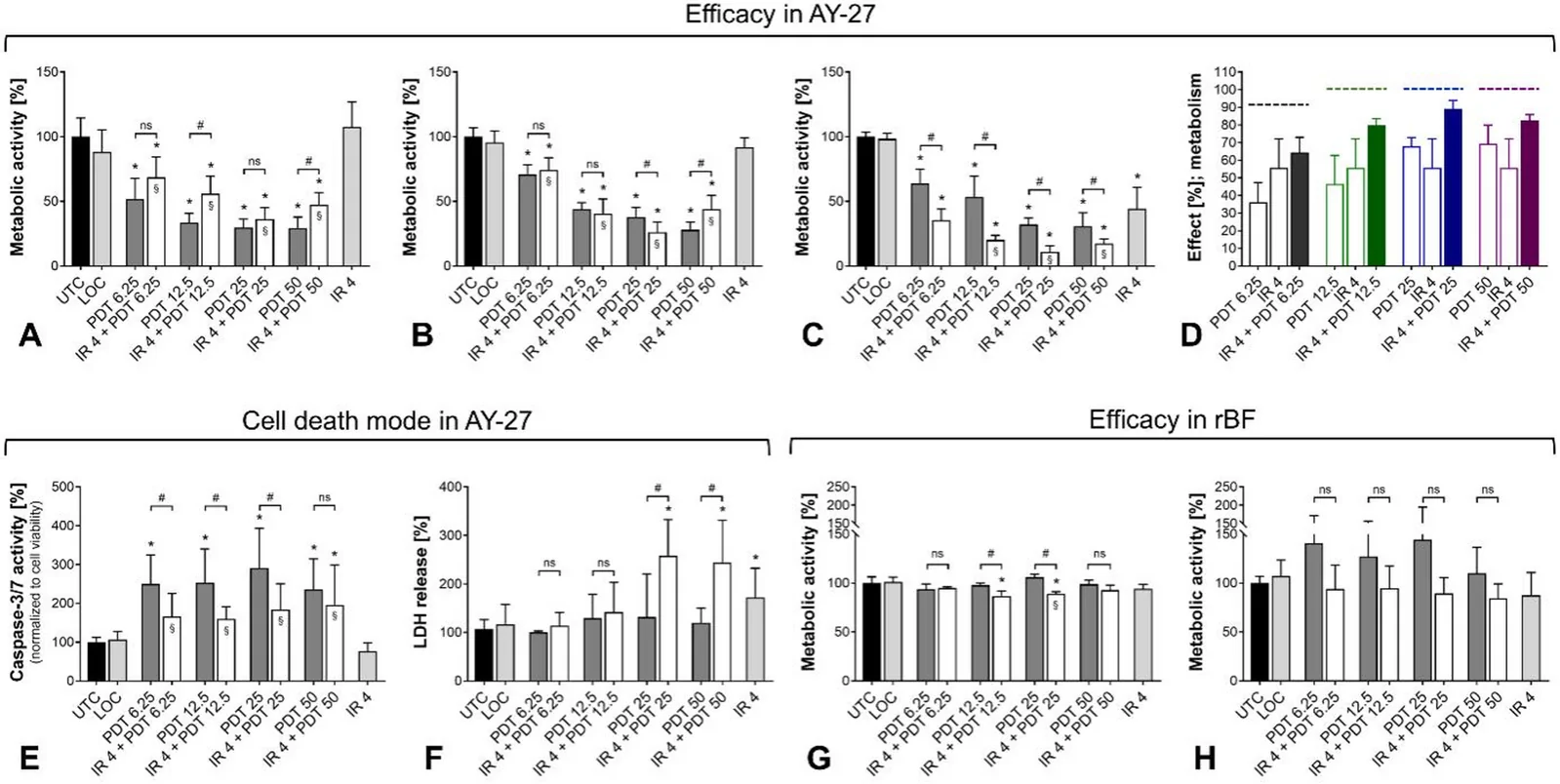
Tumor-selective, subadditive cytotoxicity of PDT with/without prior IR in cultured AY-27 cells. (**A-C**) Concentration-dependent cytotoxicity in AY-27 cells. Metabolic activity by CellTiter-Blue^®^ Cell Viability Assay 6 h (**A**), 24 h (**B**), and 72 h (**C**) after treatment using THPTS-PDT (0–50 µM THPTS; 10 J/cm^2^) ± prior IR (4 Gy). (**D**) Response additivity analysis of cytotoxicity in AY-27 after 72 h. Dashed lines indicate the expected effects (sum of E_PDT_ and E_IR_; black: IR 4 + PDT 6.25; green: IR 4 + PDT 12.5; blue: IR 4 + PDT 25; violett: IR 4 + PDT 50) of therapy combination. Combinational therapy showed subadditive effects in cultured AY-27 after 72 h. (**E**,** F**) Cell death mode in AY-27 cells. Apoptosis via Caspase-3/7 activity by Apo-ONE^®^ Homogeneous Caspase-3/7 Assay (**D**), and LDH release by CytoTox-ONE™ Homogeneous Membrane Integrity Assay (**E**) 6 h after treatment with THPTS-PDT ± prior IR; caspase-3/7 activity was normalized to cell viability (number of metabolically active cells). (**G**,** H**) Cytotoxicity in primary rBF. Metabolic activity by CellTiter-Blue^®^ Cell Viability Assay 24 h (**G**), and 72 h (**H**) after treatment using THPTS-PDT (0–50 µM THPTS; 10 J/cm^2^) ± prior IR (4 Gy). * Significant differences compared to untreated control (UTC); § significant differences between IR monotherapy and IR + PDT, *p* ≤ 0.05, One-way ANOVA, Dunnett´s multiple comparison test; #, ns significances between PDT monotherapy and IR + PDT, *p* ≤ 0.05, Mann-Whitney test. Mean + SD; *n* = 3

**Fig. 3:**
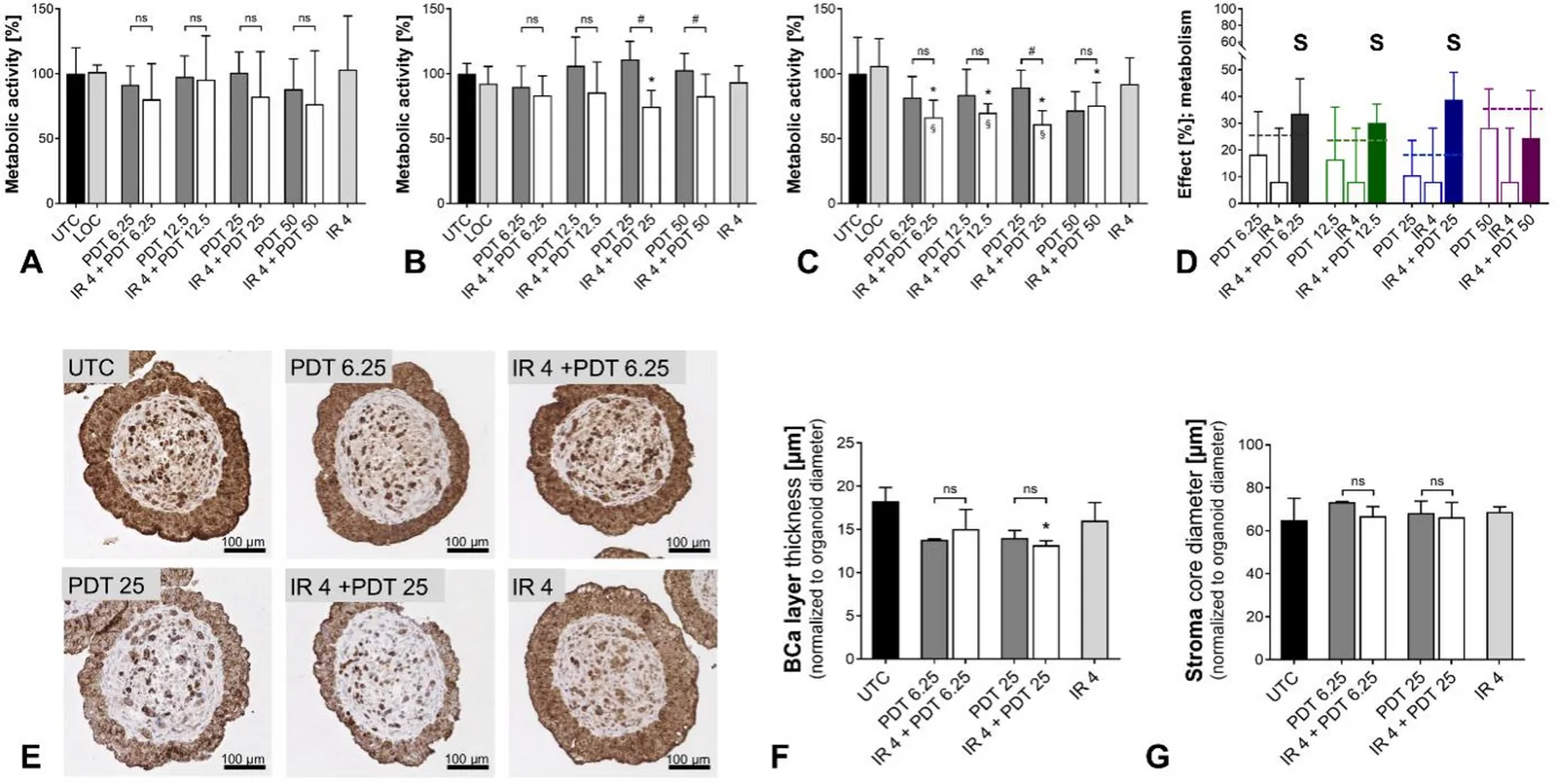
Tumor-selective, synergistic cytotoxicity of PDT with/without prior IR in AY-27/rBF spheroids. (**A-C**) Concentration-dependent cytotoxicity. Metabolic activity by CellTiter-Glo^®^ 3D Cell Viability Assay 6 h (**A**), 24 h (**B**), and 72 h (**C**) after treatment using THPTS-PDT (0–50 µM THPTS; 10 J/cm^2^) ± prior IR (4 Gy). (**D**) Response additivity analysis of cytotoxicity after 72 h. Dashed lines indicate the expected effects (sum of E_PDT_ and E_IR_; black: IR 4 + PDT 6.25; green: IR 4 + PDT 12.5; blue: IR 4 + PDT 25; violett: IR 4 + PDT 50) of therapy combination. Combinational therapy showed synergistic effects (S) for IR 4 + PDT 6.25/ PDT 12.5/ PDT 25 after 72 h. (**E-G**) Histological evaluation of treated spheroids revealed significant reduction of the tumor cell layer by IR 4 + PDT 25, while the stroma core diameter was not affected by the treatment. * Significant differences compared to untreated control (UTC); § significant differences between IR monotherapy and IR + PDT, *p* ≤ 0.05, One-way ANOVA, Dunnett´s multiple comparison test; #, ns significances between PDT monotherapy and IR + PDT, *p* ≤ 0.05, Mann-Whitney test. Mean + SD; *n* = 3

Concentration-dependent cytotoxicity and cell death mechanisms were analyzed after IR (4 Gy) and/or PDT with THPTS (0, 6.25, 12.5, 25, 50 µM) in 2D-cultured AY-27 cells (Fig. 2) and AY-27/rBF spheroids (Fig. 3). In 2D cultures, decreased viability of IR + PDT-treated cells after 6 h seemed to result from PDT only, as IR monotherapy showed no short-term effects (Fig. 2, A). PDT with prior IR led to mixed effects after 24 h, whereas a significant dose-dependent decrease of AY-27 survival resulting in up to 90% reduced cell viability was found 72 h after treatment (IR 4 + PDT 25) (Fig. 2, B, C). Effects of combination therapy were significantly higher compared to PDT and IR monotherapies. Response additivity analysis revealed subadditive effects at all concentrations (Fig. 2, D). Caspase-3/7 activation was significantly induced 6 h after PDT monotherapy, but not after IR + PDT in cultured AY-27 cells (Fig. 2, E). In contrast, release of LDH, which indicates loss of membrane integrity, increased significantly by IR + PDT (not by PDT only) within 6 h post treatment (Fig. 2, F).

In non-malignant rBF, IR + PDT resulted in a short-term decrease in cell viability at 24 h (Fig. 2, G). However, by 72 h post-treatment (Fig. 2, H), cell viability was no longer significantly different from that of the untreated control. Cellular effects in 2D culture were comparatively analyzed in 3D rat AY-27/rBF spheroids. Co-culturing of AY-27 cells and rBF in 96-well ULA plates resulted in self-organization of bladder-like spheroids which consisted of an outer tumor cell layer around an inner fibroblast core bearing single AY-27 cell nests (as recently described for human BCa organoids [29]). We performed single-dose IR (4 Gy) followed by PDT (0–50 µM THPTS; light dose 10 J/cm^2^) on day 3 post spheroid construction. After different periods of time, we analyzed viability and morphology. PDT with prior IR led to a dose-independent significant decrease of AY-27 spheroid viability resulting in 25% loss of metabolic activity after 24 h, and 39% reduction 72 h following therapy (using IR 4 + PDT 25). Single-time monotherapies showed no significant effect on metabolic activity of AY-27/rBF spheroids (Fig. 3, A-C). Response additivity analysis revealed synergism of combined treatment 72 h after IR 4 + PDT 6.25/ PDT 12.5/ PDT 25 (Fig. 3, D).

The underlying mechanisms were investigated by assessing 4-HNE, p-H2A.X, and c-Casp3 in histologically processed AY-27/rBF spheroids at early time points (2 and 16 h) following treatment with IR 4 + PDT 25. The results obtained after 16 h are presented in Additional file 3. PDT monotherapy induced a pronounced increase in 4-HNE in the tumor cell layer, whereas IR alone increased p-H2A.X levels in both the tumor cells and, to a lesser extent, the stromal core. The combined IR + PDT treatment resulted in strongly elevated levels of both markers, particularly in the tumor cell layer. Activated caspase-3 was not detected following PDT, either alone or preceded by IR (Additional file 3). Tumor-selective effects were further confirmed by histology of AY-27/rBF spheroids, immunostained for CK AE1/AE3 (AY-27, brown) 72 h after therapy. The treatment with IR 4 + PDT 25 led to a significant reduction of tumor layer thickness. In contrast, there was no effect on diameter of the non-malignant stromal cell core (Fig. 3, E-G).

### Therapeutic efficacy in vivo

A preliminary study was conducted to assess the long-term outcome of single-time PDT (PDT: *n* = 12; vs. UTC: *n* = 12) (Additional file 4). The transurethral single-time PDT (100 µM THPTS; 10 J/cm^2^) resulted in significantly shorter survival of rats in the PDT group versus untreated controls (mOS: 46 d vs. 68.5 d; *p* = 0.0222) (Additional file 4, B). The histological evaluation revealed that the PDT-treated and untreated bladders beared tumors of similar size at the time of termination (Additional file 4, C, D). To enhance treatment efficacy, multiple PDT sessions were administered in the main trial, both as monotherapy and in combination with prior IR. In a pre-trial cohort for IR dose determination (Additional file 5), fractionated irradiation with 3 × 8.5 Gy at one week intervals led to a significant prolonged survival during the trial period (Additional file 5, B). However, 80% of IR-treated rats still had large viable tumors (max. basal diameter: 1553 ± 1575 μm; vs. 2862 ± 1935 μm in UTC group; ns, *p* = 0.3152) at the end of the preliminary study period (Additional file 5, D). Tumor-adjacent healthy tissue or IR-treated pT0 bladders showed no signs of radiation-induced damages (e.g. edemia, endothelial cell loss) (Additional file 5, C). Consequently, this fractionated IR dose was used in combination with the triple-time PDT (100 µM THPTS; 10 J/cm^2^) in the main trial.

#### Enhanced survival of F344 rats after PDT with/without prior IR

**Fig. 4:**
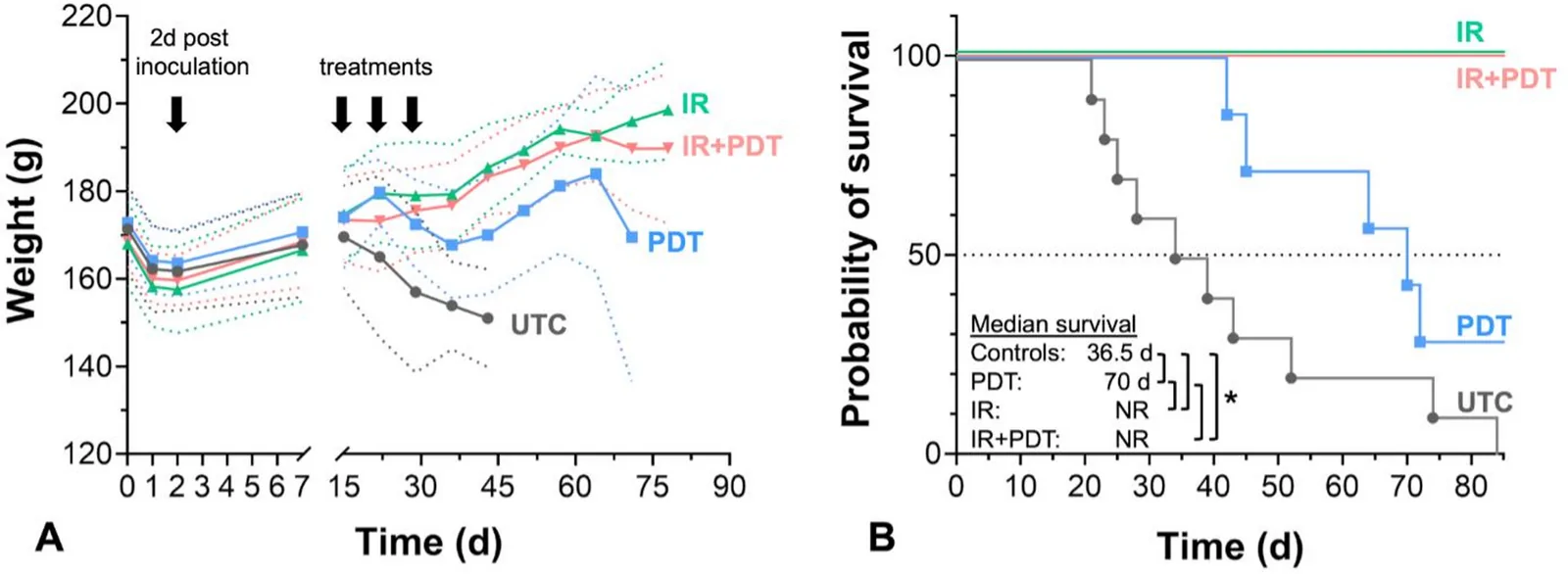
Monitoring of trial success of multiple PDT with/without prior IR. Weight development and overall survival. (**A**) Development of animal weight in different treatment groups after triple-time treatment; weight (mean ± error (dashed lines)) was monitored until median survival of the experimental group was reached. (**B**) Overall survival until reaching the termination criteria; or twice the survival time of untreated controls. Animals in the IR- and IR + PDT-treated group did not meet the termination criteria (median survival not reached, NR). * Significant differences between treatment groups, *p* ≤ 0.05, Gehan-Breslow-Wilcoxon test; *n* = 10 (UTC), 7 (PDT), 8 (IR), 8 (IR + PDT)

In total, 40 F344 rats were inoculated with AY-27 BCa cells for the main trial. We observed a weight reduction of all rats 2 d after tumor inoculation, which then mostly recovered until the first round of therapy. Beginning one week after the third treatment, the IR- and IR + PDT-treated groups showed further weight increases, whereas in PDT-treated and untreated animals weight and health declined (Fig. 4, A).

Four rats died during the first therapy from aspiration pneumonitis (due to bile) during inhalation anesthesia (*n* = 2 (PDT), *n* = 1 (IR), *n* = 1 (IR + PDT)), three animals had to be euthanized due to poor health status (*n* = 1 (PDT), *n* = 1 (IR), *n* = 1 (IR + PDT)), and 33 rats received treatment as planned. In summary, 15/33 rats had to be euthanized because of tumor-driven human endpoints and 18/33 survived without clinical symptoms. The trial was terminated 86 d after tumor induction (approx. 2-time median survival time of UTC). In Kaplan-Meier analyses, triple-time PDT showed significantly prolonged survival compared to untreated BCa-bearing animals (mOS: 70 d vs. 36.5 d; *p* = 0.0338). In the IR-treated and combinatorial therapy groups, the termination criteria were not reached during the observation period. IR + PDT led to a significant increase in median survival compared to PDT monotherapy (mOS: NR vs. 70 d; *p* = 0.0047); compared to IR monotherapy, the addition of PDT had no effect on survival. Histopathological assessment was perfomed to further differentiate between the groups (Fig. 4, B).

#### Histologically assessed tumor regression by PDT with prior IR

**Fig. 5:**
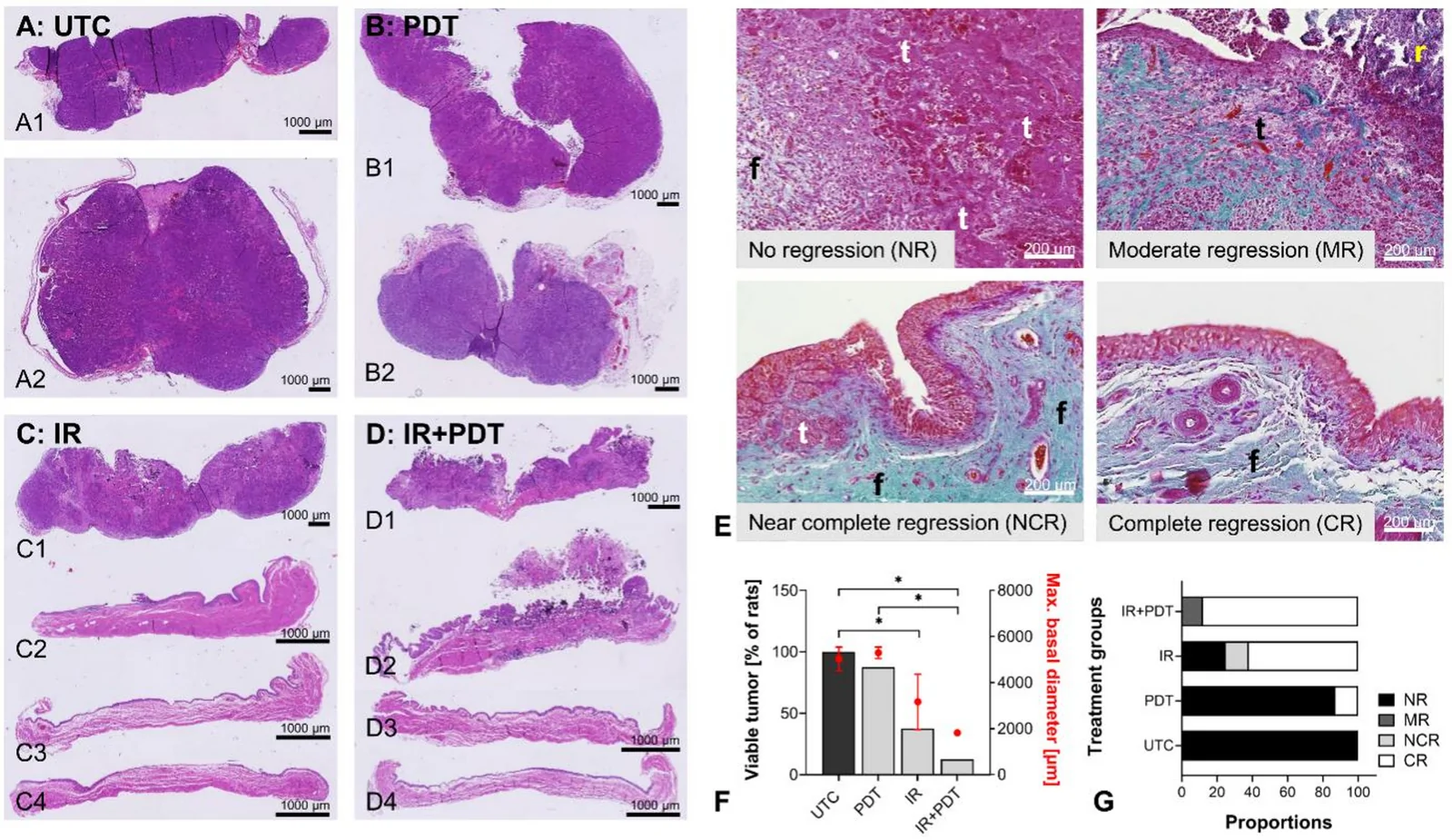
Histopathological evaluation. (**A-D**) Representative images of HE-stained rat bladders under different treatment conditions. (**A**,** B**) All untreated and most of PDT-treated animals showed partially necrotic tumors infiltrating the perivesicular fat tissue. (**C**,** D**) IR-/IR + PDT-treated bladders showed different responses ranging from large tumors (C1) to histologically normal bladder walls. (**E**) Representative images of Crossmon´s trichrome-stained bladder tumors corresponding to different TRG: dominant tumor masses with some regression zones (NR), fibrotic changes with few tumor cells or groups (MR), singular islets of tumor cells (papillary) invading the connective tissue (NCR), absence of tumor cells (CR); f, fibrosis (green); r, regression zone; t, tumor. (**F**,** G**) Quantification of tumor responses. Viable tumor count, max. basal diameter (**F**) and proportions of tumor regression grades (**G**) within different treatment groups; * significance between treatment conditions; *p* ≤ 0.05; Fisher´s exact test (single comparison); *n* = 10 (UTC), 7 (PDT), 8 (IR), 8 (IR + PDT)

Bladders were extracted and evaluated by macroscopy and histology. On dissection, bladders were covered by intraperitoneal fat and no perforations were observed. Further, there was no macroscopic evidence of abscesses in the kidneys suggesting no serious local infections. Bladder processing allowed histopathological evaluation including: (i) presence of viable tumors, (ii) maximum basal diameter of tumors, (iii) pT classification, and (iv) tumor regression grade (TRG) (Additional file 6, Fig. 5). Representative HE stains for the different treatment conditions are presented in Fig. 5, A-D. Untreated controls and nearly all PDT-treated bladders showed massive (partially necrotic) pT3 tumors with infiltrations into the perivesicular fat tissue and inflammation at the invasive margin. In the IR monotherapy and the IR + PDT group, we detected both large tumors with recognizable regression zones (Fig. 5, C1) and pathologically normal urinary bladders (Fig. 5, C3, C4 D3, D4). The quantitative analysis demonstrated that the triple-time PDT-treated bladders exhibited similar-sized tumors compared to the untreated controls, but after twice the survival time. IR monotherapy (*p* = 0.0008) and IR + PDT (*p* = 0.0007) both led to a significant reduction (vs. UTC) in viable tumor count. Compared to PDT monotherapy, combination therapy showed significant effects on tumor number (*p* = 0.0051), whereas IR alone had no significant effects (*p* = 0.0594). In addition, an advantage of combination therapy over IR monotherapy was demonstrated with regard to the number of tumors (1/8 vs. 3/8 viable tumors) and tumor size (max. basal diameter: 1817 μm vs. 3161 ± 2048 μm) (Fig. 5, F).

The tumor regression scoring was based on previously published criteria which are listed in Table 3 [26]. Crossmon’s trichrome stainings of the different regression levels are exemplarily shown in Fig. 5, E. The scoring allowed further differentiation of the IR and IR + PDT group, and showed that only the triple-time IR + PDT combination therapy led to a response in all bladder tumors (no responders: 0/8 (IR + PDT) vs. 2/8 (IR)) (Fig. 5, G). Thereby complete regression was achieved in 7/8 rats after IR + PDT treatment. Potential side effects of IR + PDT in bladders exhibiting complete tumor regression are presented in Additional file 7. In approximately half of the treated rat bladders, moderate histopathological changes in the bladder wall were observed, including urothelial damage and thinning, edema, fibrosis, and inflammation.

#### Local immune stimulation by PDT with/without IR

**Fig. 6:**
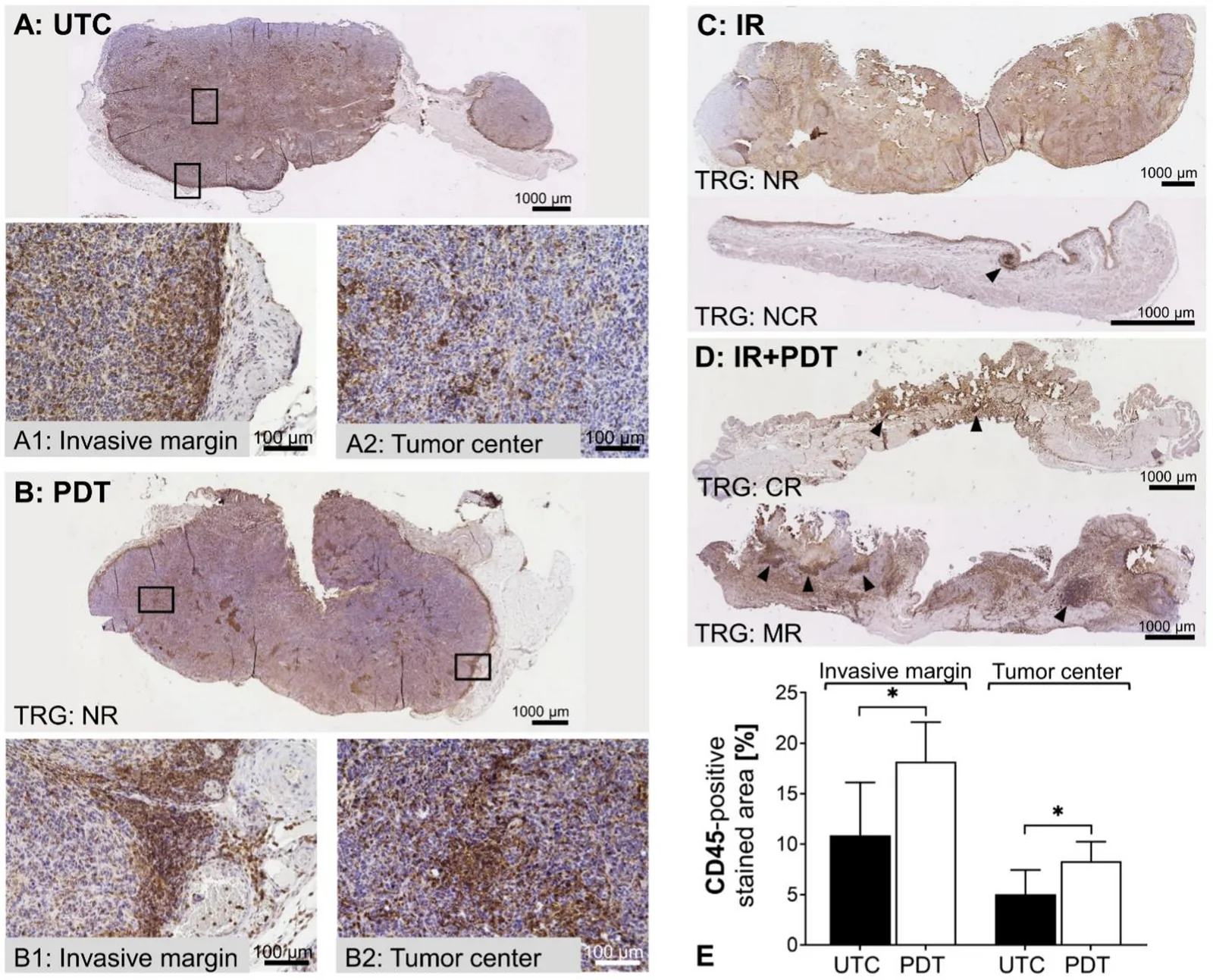
Evaluation of local immune cell recruitment. (**A-D**) Representative images of anti-CD45 immunostained rat bladders after different treatment conditions and TRG. (**A**,** B**) Untreated and PDT-treated rat bladders; tumors exhibited accumulations of leukocytes at the invasive margin and partially within the tumor center. (**C**) IR-treated rat bladders; a NR tumor showed unspecific background staining of necrotic tumor regions, while the NCR tumor showed a highly postitive leukocyte nest (arrows) in the region of invading tumor cell islets. (**D**) IR + PDT-treated rat bladders; the MR tumor exhibited large numbers of strongly CD45-positive immune cells, especially underneath the tumor regression zones (arrows). (**E**) Quantification of immune stimulation in PDT-treated rats vs. untreated controls. Highly positive-stained regions were determined at the invasive margin and the tumor center of tumor-bearing rat bladders; * significance between treatment conditions; *p* ≤ 0.05, Mann-Whitney test, mean + SD, *n* = 10 (UTC), 6 (PDT)

To investigate if PDT and IR induced local immune responses, three different sections per bladder sample (in a distance of 100–200 μm) were immunostained for CD45 within the tumor. Representative images of rat bladders after different treatment conditions are presented in Fig. 6, A-D. Untreated and PDT-treated rat bladder tumors exhibited accumulations of strongly stained leukocytes along the invasive margin and partially within the tumor center (Fig. 6, A, B). Abundance of CD45 leukocytes was analyzed in ≥ 10 images (20 x magnification) per section (invasive margin vs. tumor center) by quantification of the highly positive-labeled area (%). Images of the tumor center served entirely as a region of interest (ROI). The invasive margin was defined as a 100 μm-wide region along the tumor border (= ROI). Significantly increased numbers of CD45-positive leukocytes were detected at the invasive margin as well as the tumor center after PDT monotherapy compared to untreated controls (invasive margin: *p* = 0.0124; tumor center: *p* = 0.0155) (Fig. 6, E). CD3 immunohistology to investigate adaptive immune stimulation showed a slightly enhanced abundance of CD3-positive T cells after PDT (versus UTC), although the increase was not significant (Additional file 8). Different results were seen in IR-/IR + PDT-treated groups. Non-tumor bladders showed no immune cell accumulations, but only individually distributed leukocytes. The IR-treated rat bladder showing near-complete regression exhibited highly positive leukocyte groups in the region of invading tumor cell nests. Further, the IR + PDT-treated tumor exhibited large numbers of strongly positive CD45-positive immune cells, especially underneath the tumor regression zones (Fig. 6, C, D). A statistical analysis in the IR monotherapy and combination therapy trial groups was not possible due to frequently observed complete tumor regression.

## Discussion

Focal therapies such as photodynamic therapy and radiotherapy are promising options for organ-sparing treatment of urothelial carcinoma. The advantage of a combined application of PDT (e.g. using temoporfin, palladium-bacteriopheophorbide) and radiotherapy has already been demonstrated for various tumor entities [18]. In the present study, we aimed to demonstrate tumoricidal effects in the orthotopic rat BCa model. Investigations on synergistic and tumor-selective cytotoxicity were performed in 2D cultured AY-27 BCa cells and spheroids of F344 rats. Treatment with PDT with/without prior IR resulted in significant reduction of metabolic activity even 6 h post therapy, which indicated induction of stressful growth arrest by PDT monotherapy. THPTS-PDT monotherapy led to early onset of apoptosis, whereas IR + PDT mainly provoked loss of membrane integrity in AY-27 cells. A significant advantage of PDT with prior IR was observed after 72 h compared to PDT monotherapy over the whole dose range, whereas primary rBF were not affected by the treatment. Response additivity analysis revealed that IR + PDT had subadditive but not additive/synergistic effects in cultured AY-27 cells. Although the effects of IR + PDT on metabolic activity were less pronounced in AY-27/rBF spheroids, likely due to the well-documented increased resistance of 3D cell cultures to anti-tumor therapies [30], synergistic effects of the combined treatment were observed at most concentrations. These findings may be explained by the principle that two individually semi-lethal treatments can exert a lethal effect when combined, thereby resulting in synergism [21]. This is consistent with our previous study, in which we demonstrated synergistic effects of IR + PDT in human RT-112 organoids [19], further highlighting the suitability of organotypic 3D cultures for the in vitro evaluation of novel therapeutic approaches [29, 31]. Cytotoxic mechanisms of IR + PDT were subsequently investigated in histologically processed spheroid samples. Notably, only the combined IR + PDT treatment resulted in significantly increased levels of both oxidative stress and DNA damage especially in the spheroid periphery, further supporting the therapeutic benefit of the combined approach. Accordingly, we detected a slight but significant reduction of the outer AY-27 tumor cell layer, but not the stromal core, further supporting the hypothesis of tumor-selectivity. In our preliminary in vivo study (PS I), a single treatment with THPTS-PDT unexpectedly reduced overall survival in the experimental group. Single-fraction PDT may be less effective than fractionated treatment regimens, as a single illumination can lead to rapid oxygen depletion and insufficient generation of reactive oxygen species (ROS), potentially leaving residual tumor cells capable of activating survival signaling pathways [32]. These findings suggest that fractionated or multi-session PDT may provide more sustained treatment efficacy and improve long-term tumor control. Therefore, repetitive treatment regimens resembling current clinical standards were realized in our main in vivo studies.

The bladder is easily accessible via the physiological entrance throuth the urethra, making BCa suitable for minimal-invasive treatment. Local PDT was performed by topical THPTS instillations into the rat bladder, followed by illuminations via a transurethrally inserted glas fibre 14/21/28 d after tumor inoculation. Overall, treatments were well-tolerated and 33/40 animals were included in the analyses. Triple-time PDT monotherapy led to a doubling of median survival but not to sustained tumor regression compared to untreated control animals. The interpretation of our results in the light of existing scientific literature is demanding, due to varying disease models (xenograft vs. orthotopic BCa models) or treatment conditions (vascular-targeted vs. intravesical PDT; different time periods between tumor inoculation and PDT; single vs. multiple applications) used in other studies. Single-time, vascular-targeted PDT using WST11 (Tookad^®^, performed 12 d post tumor inoculation) led neither to increased survival nor to significant tumor reduction in a mouse BCa xenograft model [33]. In a subsequent study, Rosenzweig et al. proved an evolving immune reaction at the tumor site with accumulation of CD3-positive T cells after vascular-targeted WST11-PDT, which resulted in increased survival (+ 42% mOS) in the same mouse model [34]. In comparison, similar WST11-PDT (performed 6 d post tumor inoculation) led to significant tumor reduction detected after 10 d, and to a 50% increase of median survival in an orthotopic mouse model [35]. Moreover, Lamy et al. observed near complete tumor regression in 31% of animals 30 d after intravesical PDT using Hexyl 5-aminolevulinate on day 5 post tumor inoculation in the F344 Fischer rat model. Survival data were not examined in the study [36]. To our best knowledge, this is the first study showing a 100% increase of median survival by local triple-time PDT monotherapy of BCa in vivo.

Focal therapies such as PDT can induce stimulation of the host´s immune system [6–8]. As shown in orthotopic BCa models, both vascular-targeted and intravesically applied PDT could lead to significantly increased immune cell counts (T cells, natural killer cells, dentritic cells) and/or changes of immune cell localization from the tumor periphery to tumor center [35, 36]. Here, tumors treated by THPTS-PDT exhibited a general immune response with significantly enhanced CD45-positive leukocyte accumulations at the invasive margin and the tumor center. Enhanced levels of CD3-postitive T cells representing adaptive immunity after PDT were not detected in our study, which might be due to different time intervals after tumor inoculation caused by nearly doubled median survival in the UTC and PDT group. During this time period, T cell dysfunction, exhaustion or tolerance may occured, thus contributing to diminished PDT long-term effect. Indeed, PDT-treated bladders showed massive pT3 tumors similar to the UTC group. The triple-time combination therapy with IR, on the other hand, led to almost complete tumor remission. This is in accordance with previous reports demonstrating the advantage of combined application of PDT and radiotherapy for various cancer types in clinical studies [18].

Although multimodal treatments based on photodynamic therapy have been increasingly explored, several challenges limit their clinical application: [1] the difficulty of achieving targeted PS delivery to deep regions of tumor tissue; [2] the challenge of achieving complete and sufficient tumor illumination while minimizing light-induced tissue fibrosis; and [3] the immunosuppressive tumor microenvironment, which can limit therapeutic efficacy. Indeed, imaging the tumor accumulation and penetration of THPTS remains challenging due to its chemical properties, including its non-fluorescent nature and high sensitivity to visible light. To date, the therapeutic depth achieved with in vivo THPTS-PDT has primarily been assessed by visualizing the proportion of necrotic tissue within treated solid tumors [11]. Systemic administration of THPTS via intravenous injection, enabling its delivery to deeper tumor regions through the tumor vasculature, followed by localized laser irradiation, may provide a feasible approach to overcome this limitation. High-power near-infrared lasers (e.g., 10–15 W) have been shown to penetrate living tissues to considerable depths [37], suggesting that they could be suitable for this application. A further limitation of our study concerns the determination of the applied IR dose. BED calculations and α/β ratios are based on reference values [23, 24] from the literature and have not been specifically determined for AY-27 cells or normal rat bladder cells. In addition, our study lacks a control group in which the therapy is administered to healthy, tumor-free rats, which would allow us to assess potential off-target toxicity. Future research will focus on optimizing the treatment regimen for the RT + PDT combination therapy, including dose adjustment and optimization of light application techniques. A possible solution for this involves the use of alternative excitation sources (unaffected by tissue thickness) such as chemo-/bioluminescence or X-rays, which are transduced into light photons to activate nearby PS by so-called scintillating nanoparticles (NPs) [18, 38]. For instance, a near-infrared PS (pyrene-fused (bi-) phenaziothiadiazoles) was encapsuled into DSPE-mPEG_2000_ NPs. The PS was activated by aggregation-induced emission, and efficiently provoked tumor reduction in an orthotopic BCa mouse model [39]. Further, He et al. engineered tumor-homing peptide-labeled nanotransducers. Activation by X-rays resulted in enhanced tumor regression, recurrence inhibition and restored immune homeostasis in mice with autochthonous bladder tumors [40]. NPs that emit light at the wavelengths corresponding to the THPTS absorption spectrum could represent another potential approach. Using PSs capable of mediating less oxygen-dependent type I reactions might help to overcome challenges of inefficient ROS generation due to rapid oxygen consumption in solid tumors which can limit the efficacy of IR.

In summary, multimodal focal therapies based on PDT and IR could enable an effective organ-sparing treatment option for bladder carcinoma. Combinations with immune cell-targeted therapies (e.g. immune checkpoint inhibitors), which break the immunsuppressive interactions between cancer and immune cells, may enhance the inherent immunogenic effects of focal therapies [41, 42].

## Conclusions

In this study, combinatorial treatment of bladder tumors by IR and near-infrared PDT was more effective than corresponding single treatments while showing no severe side effects. The dual treatment might be important for patients having non-muscle-invasive BCa without metastasis, especially in high-grade non-muscle-invasive BCa (pT1G3) where bladder removal is recommended. However, even patients with non-metastatic muscle-invasive BCa (up to 15 mm tumor thickness) could benefit from this treatment option. Further invesigations of the local or even systemic immune stimulation could provide more information about possible treatment modalities.

## Supplementary Information

Supplementary Material 1

Supplementary Material 2

Supplementary Material 3

Supplementary Material 4

Supplementary Material 5

Supplementary Material 6

Supplementary Material 7

Supplementary Material 8

## Data Availability

All data generated or analyzed during this study are presented in this published article or in the supplementary material. Additional information about data generation or processing are available on request from the corresponding author.

## Abbreviations

BCa: Bladder cancer
BED: Biologically effective dose
NMIBCa: Non-muscle-invasive bladder cancer
MIBCa: Muscle-invasive bladder cancer
PDT: Photodynamic therapy
PS: Photosensitizer
THPTS: Tetrahydroporphyrin-tetratosylate
ROS: Reactive oxygen species
IR: Ionizing radiation
rBF: Rat bladder fibroblasts
UTC: Untreated control
LOC: Light only control
LDH: Lactate dehydrogenase
mOS: Median overall survival
TRG: Tumor regression grade
NR: No or minimal regression
MR: Moderate regression
NCR: Near complete regression
CR: Complete regression
