# Supplementary material for: Enhanced survival through repeated photodynamic therapy and almost complete tumor regression by prior radiation therapy in an orthotopic rat bladder cancer model
Source: Cancer Cell Int. 2026 Sep 17;26:315. doi: 10.1186/s12935-026-04465-2 (PMC13587558; doi:10.1186/s12935-026-04465-2)
Supplement: Supplementary file 1 [file 12935_2026_4465_MOESM8_ESM.pdf]

Table S6: Data of in vivo study obtained from histopathological evaluation.

Group	Rat no.	OS (d)	Viable tumor	Max. basal diameter (µm)	pT stadium	TRG	Regression zone	Inflammation
UTC	1	74	1	4952	3	NR	n.a.	3
	2	28	1	3628	3	NR	n.a.	2
	3	25	1	4364	3	NR	n.a.	2
	4	52	1	6573	3	NR	n.a.	3
	5	23	1	3761	3	NR	n.a.	3
	6	84	1	5036	3	NR	n.a.	2
	7	39	1	3848	3	NR	n.a.	1
	8	43	1	7977	3	NR	n.a.	3
	9	34	1	6578	3	NR	n.a.	2
	10	21	1	3619	3	NR	n.a.	2
PDT	1	72	1	4481	3	NR	1	3
	2	86	1	6494	3	NR	0	3
	3	70	1	5687	3	NR	1	3
	4	86	0	n.a.	0	CR	1	0
	5	64	1	5263	3	NR	0	2
	6	45	1	4905	3	NR	0	2
	7	42	1	4925	3	NR	0	2
IR	1	86	1	213	1	NCR	0	1
	2	86	0	n.a.	0	CR	0	0
	3	86	0	n.a.	0	CR	0	0
	4	86	0	n.a.	0	CR	0	0
	5	86	0	n.a.	0	CR	1	0
	6	86	1	4613	3	NR	1	3
	7	86	1	4656	3	NR	1	3
	8	86	0	n.a.	0	CR	1	0
IR+PDT	1	86	1	1	2	MR	1	3
	2	86	0	0	0	CR	0	0
	3	86	0	0	0	CR	0	2
	4	86	0	0	0	CR	0	0
	5	86	0	0	0	CR	1	0
	6	86	0	0	0	CR	1	0
	7	86	0	0	0	CR	1	0
	8	86	0	0	0	CR	1	0
